# P-1332. Activity of aztreonam-avibactam tested against genetically characterized non-carbapenemase producing carbapenem-resistant Enterobacterales

**DOI:** 10.1093/ofid/ofaf695.1520

**Published:** 2026-01-11

**Authors:** Mariana Castanheira, John Kimbrough, Dmitri Debabov, Helio Sader

**Affiliations:** Element, North Liberty, IA; Element Iowa City (JMI Laboratories), North Liberty, Iowa; Abbvie, Irvine, California

## Abstract

**Background:**

Carbapenem-resistant Enterobacterales (CRE) isolates are considered a threat to human health. While most of the CRE isolates produce carbapenemases, a portion of them have combinations of resistance mechanisms that elevate the carbapenem MIC values along with other β-lactams. We evaluated the activity of aztreonam-avibactam (ATM-AVI) and comparator agents against 111 non-carbapenemase producing (non-CPE) CRE collected over a 7-year period in US hospitals.

**Figure:**
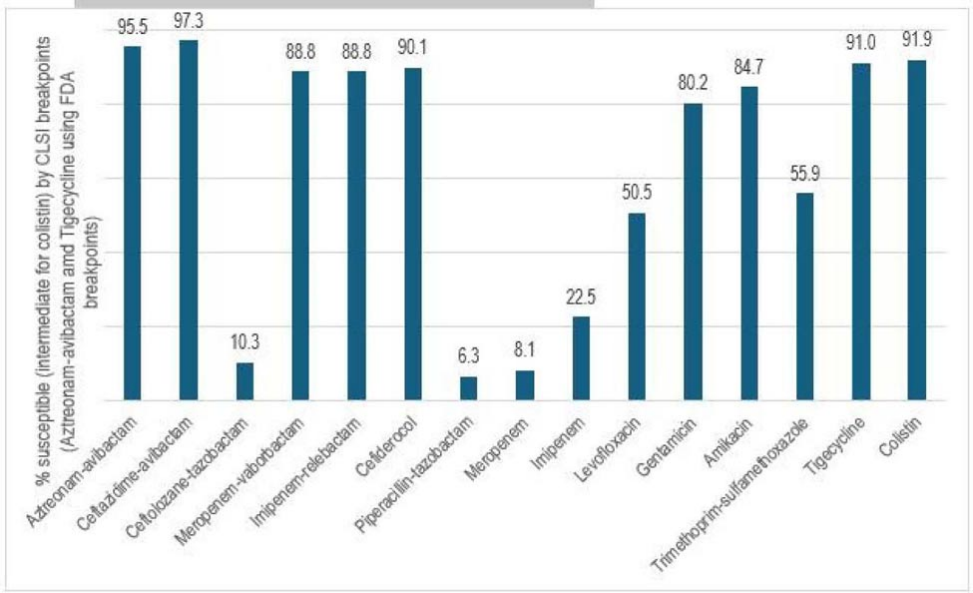
Acitivty of aztreonam-avibactam and comparator agents

**Methods:**

A total of 72,265 Enterobacterales isolates were collected during 2016-2023 in US hospitals. Susceptibility testing was performed by the CLSI reference broth microdilution method. CLSI and FDA interpretative criteria were applied. CRE isolates were submitted to whole genome sequencing and evaluated for the presence of β-lactamases. Non-CPE-CRE were further examined for genetic mechanisms underpinning β-lactam resistance.

**Results:**

Among 694 CREs, 111 (16.0% of the CRE, 0.2% overall) were non-CPE. Most isolates were *K. pneumoniae* (KPN, n=35) and *K. aerogenes* (KAR, n=30) but also 8 other genera. Isolates were distributed in all US Census divisions. 29/35 KPN and 10/13 *E. coli* isolates carried CTX-M with most being CTX-M-15. Acquired enzymes were not common among other species. Early terminations/lost start or stop codons in outer membrane protein (OMP) genes were noted in 64 isolates for a single gene and in 27 for both *ompC/ompK36* and *ompF/ompK35*. KAR (6/30 isolates), *C. freundii* (1/3) and *E. cloacae* species complex (6/17) displayed RamR disruptions. ATM-AVI and ceftazidime-avibactam were active against 95.5% and 97.3% of the isolates and had the highest susceptibility rates among the agents tested (Figure). ATM-AVI resistant isolates were one *E. coli* producing CTX-M-15 and CTX-M-33 with AcrR, OMP, and PBP3 disruptions and one KAR with OMP and RamR disruptions.

**Conclusion:**

Carbapenem resistance among non-CPE-CRE isolates is complex and involves β-lactamase production with reduced access to the bacterial target due to changes in OMP and regulators that might increase drug efflux. Despite the combinations of resistance mechanisms, ATM-AVI and ceftazidime-avibactam displayed good activity against these isolates that were resistant to other agents.

**Disclosures:**

Mariana Castanheira, PhD, Melinta Therapeutics: Advisor/Consultant|Melinta Therapeutics: Grant/Research Support Helio Sader, United States Food and Drug Administration: FDA Contract Number: 75F40123C00140

